# Clonal hematopoiesis and biological aging: an interaction that increases cancer risk?

**DOI:** 10.1093/jnci/djag309

**Published:** 2026-10-03

**Authors:** Jake Tobin, George S Vassiliou

**Affiliations:** Cambridge Stem Cell Institute, University of Cambridge, Cambridge, United Kingdom; Department of Haematology, University of Cambridge, Cambridge, United Kingdom; Department of Haematology, Cambridge University Hospitals NHS Foundation Trust, Cambridge, United Kingdom; Cambridge Stem Cell Institute, University of Cambridge, Cambridge, United Kingdom; Department of Haematology, University of Cambridge, Cambridge, United Kingdom; Department of Haematology, Cambridge University Hospitals NHS Foundation Trust, Cambridge, United Kingdom

Clonal hematopoiesis (CH) refers to the clonal expansion of a hematopoietic stem cell and its progeny. When CH expands to a variant allele fraction ≥2% in whole blood DNA, the phenomenon is arbitrarily defined as CH of indeterminate potential (CHIP).^1^ CHIP has attracted significant research interest due to its association with an increased risk of myeloid malignancies, as well as nonhematological diseases including several types of solid cancers.^2-4^ Predictive tools have been developed to quantify the risk of developing myeloid cancers among CH/CHIP carriers,^5^^,^^6^ but there are no such tools for solid cancer risk.

CHIP becomes increasingly common with advancing age; however, it is now accepted that chronological age is an imperfect measure of functional aging in many tissues.^7-9^ It is also clear that common genetic variation influences the risk of developing CH/CHIP.^4^ In addition, we now understand that individuals with an increased genetic predisposition to CH also display epigenetic age acceleration,^4^ and that the CH clonal expansion rate is associated with genetically predicted and measured epigenetic clocks.^10^ Furthermore, hematological/immunological phenotypes such as myeloid differentiation bias and inflammation are associated with both CH/CHIP and advancing age.^4^^,^^11^^,^^12^ However, despite these links, CH/CHIP and biological aging are independently influenced by many different genetic and nongenetic factors and are not co-linear.^13^ It is also worth noting that biological aging can be measured in many different ways, and there is no formal consensus on how to incorporate this into predictive models for cancer risk.

Wang et al. report, in the *Journal of the National Cancer Institute*, the results of an investigation of the interaction between CHIP and biological aging as determinants of cancer risk.^14^ To do this, they identified individuals with mutations in any of 44 CH driver genes using Mutect2^4^ and computed biological aging scores using *PhenoAgeAccel*^15^ for 332 911 UK Biobank (UKB) participants. Using this approach, they categorize individuals into those with vs without CHIP and into biologically “older” (44.4%) and “younger” (55.6%) strata, and use these binary classifications to investigate the relationship between biological aging, CHIP, and cancer risk.

Their key findings were that older biological age interacted with CHIP to enhance the relative risk of developing hematological neoplasms and lung cancer. Although each of CHIP and biological aging were expectedly associated with elevated risk of these neoplasms, the study also reports a significant interaction between the two. Specifically, individuals with both CHIP + “older” biological age had a hazard ratio (HR) for hematological cancers of 6.56 compared with those with only CHIP (HR = 3.57) or with only “older” biological age (HR = 1.35), when compared to controls with neither. Similarly, the HR for lung cancer was 2.63 for CHIP + biologically older, vs 1.38 for CHIP only and 1.55 for biologically older. The authors then calculated relative excess risk interaction (RERI) and the attributable proportion (AP) of the risk to the double exposure and concluded that 40% of the risk of hematological neoplasia and 27% of the risk of lung cancer were directly attributable to the interaction between CHIP and older biological age. As their initial analyses were most suitable for examining additive interactions between the risks of CHIP and biological age, the authors also investigated potential multiplicative interactions for these neoplasms. These analyses revealed such an interaction between CHIP and older phenotypic age for hematological neoplasia (HR = 1.03; *P*_interaction_ < .001) but not for lung cancer.

The authors acknowledge that 1 important limitation of their analyses was the fact that *PhenoAgeAccel* is calculated using phenotypic parameters that are themselves linked to CHIP, including red cell distribution width (RDW), which carries the heaviest weight among 10 such parameters. Because a raised RDW is not only a well-known feature of CHIP,^16^ but is also associated with an increased risk of myeloid malignancies among CHIP carriers,^3^^,^^5^ it is possible that it was confounding the observed interaction between CHIP and biological aging. To examine this, the authors focused their analyses on CHIP due to *DNMT3A* or *TET2* gene mutations, because these are not usually associated with a raised RDW. This focused analysis showed that the interaction between *DNMT3A/TET2*-CHIP and biological aging was no longer significant for hematological malignancies. However, the additive interaction remained significant for *DNMT3A/TET2*-CHIP (RERI = 0.67, 95% CI 0.11 to 1.23) when lung cancer risk was considered. The authors conclude that interaction between CHIP and biological aging may represent a true synergism that increases lung cancer risk, while in the context of hematological malignancy the observed synergy with overall CHIP may have been driven by high-risk subtypes (eg, CHIP driven by *ASXL1*, *JAK2*, *SRSF2*, or *SF3B1* mutations). It is also possible that RDW and other shared determinants of both biological age and high-risk CHIP (eg, mean cell volume, CRP, chronological age) are behind the apparent interaction.^5^

Overall, Wang et al. describe hitherto unreported interactions between biological age and CHIP in determining cancer risk. This is a timely contribution to the field, as predictive risk scores for CHIP carriers are gaining increasing attention, and there is a growing recognition of complex relationships between CHIP and nongenetic factors such as epigenetic changes and inflammation.^17^ Nevertheless, several important issues raised by this study require further clarification. Foremost are the collinearity and shared determinants of high-risk CHIP and biological aging, making it difficult to quantify their true interaction in determining blood cancer risk. The fact that *DNMT3A/TET2*-CHIP in isolation additively interact with biological aging to increase lung cancer risk makes this particular observation more robust, but it will need to be corroborated in independent datasets/cohorts. In addition, these findings would be greatly strengthened if the interaction between CHIP and biological aging is replicated when using approaches to quantify biological age that are less reliant on measurements that also track high risk CHIP, such as the proteomic aging clock.^13^ Once this is achieved, it may be possible to integrate biological age into risk stratification and cancer prevention strategies as the authors suggest.

It is also noteworthy that the authors use binary categories of CHIP vs no CHIP and “young” vs “old” to conduct their analyses. Although such binary classifications can simplify analyses, they can also obscure continuous relationships and ones that become stronger or weaker at extremes. Future studies should consider this approach, particularly as current predictive tools of blood cancer risk, such as MN-Predict^5^ and CHRS,^6^ are strongly influenced by CHIP clone size.

Similarly, the authors reported the biological age metric derived from *PhenoAgeAccel* as a binary classification of “older” vs “younger,” while UKB participants existed on a continuous spectrum of *PhenoAgeAccel* values. Although this helped to simplify analyses, it is noteworthy that the authors found substantial discrepancies between the impact of *PhenoAgeAccel* (continuous) and *PhenoAgeAccel* (categorical) on disease risk (Table S4). This is illustrated by the example of myeloid malignancies, where HR estimates of continuous (HR = 9.76) and categorical (HR = 1.87) *PhenoAgeAccel* differ significantly. Therefore, before *PhenoAgeAccel* or a similar metric is incorporated into risk stratification tools, it would be important to establish whether the findings of this study are conserved or even enhanced when continuous *PhenoAgeAccel* estimates are used.

Moving forward, it is important to consider how best to leverage the findings of this study. Existing predictive tools can predict myeloid neoplasia risk with reasonable sensitivity and specificity. For example, receiver-operator curves for the Clonal Hematopoiesis Risk Score (CHRS) show an average concordance index of 0.807,^6^ while the corresponding values for MN-Predict range from 0.74 to 0.92 for different myeloid malignancies.^5^ Both MN-Predict and CHRS incorporate several of the parameters used to determine the *PhenoAgeAccel* score. It would be important to validate whether such tools are improved by the addition of a biological aging measurement and whether any improvements in performance justify additional testing to capture the latter.

Predictive tools used to estimate lung cancer risk such as PLCOm2012 and the Liverpool Lung Project (LLP) models are primarily based on a more descriptive history of personal and family risk factors.^18^^,^^19^ As laboratory results are not included in these models, it may be more difficult to seamlessly integrate biological age estimates. Also, PLCOm2012 incorporates ethnicity as an input variable, yet *PhenoAgeAccel* was only applied to White UKB participants in this study.

As a final consideration, risk prediction strategies are most effective when there is consensus on how to manage high-risk individuals. Were biological age and CHIP status to be validated as important determinants of lung cancer risk, it is likely that low-dose computed tomography scans could be offered for screening high-risk individuals. Also, individuals with high-risk CHIP can be monitored for evidence of progression toward myeloid cancers. However, to capitalize on the findings of this and other similar predictive studies, it will be vital to ensure that tolerable interventions are developed that allow us to prevent progression to neoplasia in those at high risk.

## Data Availability

No new data were generated or analyzed for this editorial.
